# Magnetization dynamics at finite temperature in CoFeB–MgO based MTJs

**DOI:** 10.1038/s41598-023-29597-7

**Published:** 2023-02-14

**Authors:** Sutee Sampan-A-Pai, Rattaphon Phoomatna, Worawut Boonruesi, Andrea Meo, Jessada Chureemart, Richard F. L. Evans, Roy W. Chantrell, Phanwadee Chureemart

**Affiliations:** 1 Department of Physics, Mahasarakham University, Mahasarakham, 44150 Thailand; 2 School of Physics, Engineering and Technology, University of York, York, YO10 5DD UK

**Keywords:** Electronic and spintronic devices, Atomistic models, Computational methods, Magnetic properties and materials

## Abstract

The discovery of magnetization switching via spin transfer torque (STT) in PMA-based MTJs has led to the development of next-generation magnetic memory technology with high operating speed, low power consumption and high scalability. In this work, we theoretically investigate the influence of finite size and temperature on the mechanism of magnetization switching in CoFeB–MgO based MTJ to get better understanding of STT-MRAM fundamentals and design. An atomistic model coupled with simultaneous solution of the spin accumulation is employed. The results reveal that the incoherent switching process in MTJ strongly depends on the system size and temperature. At 0 K, the coherent switching mode can only be observed in MTJs with the diameter less than 20 nm. However, at any finite temperature, incoherent magnetization switching is thermally excited. Furthermore, increasing temperature results in decreasing switching time of the magnetization. We conclude that temperature dependent properties and thermally driven reversal are important considerations for the design and development of advanced MRAM systems.

## Introduction

In today’s information and technology based era, magnetic tunnel junctions (MTJs) have generated significant interest for their potential applications, especially spin transfer torque-magnetic random access memory (STT-MRAM) as a non-volatile, high speed and low power consumption memory device^1,2^. STT-MRAM functionality is based on MTJs with perpendicular magnetic anisotropy (PMA) comprising an insulator sandwiched between two ferromagnets, the pinned layer (PL) with fixed magnetization orientation and the free layer (FL) with magnetization with bistable orientations. Induced spin currents in the MTJ achieve switching via spin torque at high power and detection of bit polarity via magnetoresistance at low power. A model combining magnetization dynamics with spin transport is clearly important for the understanding of basic physical processes and future device development. To design a high performance STT-MRAM for advanced integrated circuits, scaling the device size towards smaller and smaller dimensions is one of the major difficulties in MTJ development. It has been now established that a ferromagnet such as CoFeB exhibiting low damping constant and high perpendicular magnetic anisotropy (PMA) can provide high thermal stability, low critical current density and fast switching time of the magnetization reversal process^3–5^. However, to make STT-MRAM a concrete memory alternative, further study of magnetic properties and magnetization dynamic of magnetic materials related to the performance of MTJs is required.

The operating speed of STT-MRAM is highly dependent on the current density which is injected into the MTJ structure. The current becomes spin polarized by the pinned layer of the MTJ tunnels through the MgO layer and switches the free layer due to the spin torque arising from the $s-d$ exchange interaction^2,6–8^. The magnitude of STT is directly proportional to the current density delivered to the MTJ structure, affecting the switching time and reversal process of magnetization^9^. Understanding the spin torque phenomenon is critical for proper STT-MRAM designs since STT allows the magnetization direction to be reversed rapidly and with low energy consumption when compared to conventional MRAM that employs a magnetic field to reverse the magnetization.

The magnetization reversal process driven by STT in the MTJ structure has been widely studied both in experiments^3,10–12^ and simulations^10,13–15^. It is reported that the critical current density, which is the minimum current density used to reverse magnetization within a certain time interval, is around 10 MA/cm$^2$ for switching within a few nanoseconds^1,10,12^. The experimental investigation of the magnetization reversal process can be carried out by measuring the resistances of anti-parallel and parallel configurations of the MTJ stack^16–18^. The majority of theoretical studies have used micromagnetic models through the LLG equation and introduced the effect of spin torque via Slonczewski terms^19,20^. The effect of STT naturally consisting of adiabatic and non-adiabatic terms is generally introduced through coefficients $\mu$ and $\beta$ respectively, the magnitudes of both coefficients being generally considered as unknown constants. In addition, the tunneling current flowing into the MTJ structure, which depends on the relative angle between the magnetization in two ferromagnets, is assumed to be constant. The description of STT via Slonczewski approach has also been adapted to atomistic models by applying the torque to each single spin within the system and by allowing the adiabatic and non-adiabatic terms to be thickness dependent^14^. As in the micromagnetic case however, the injected current density is uniform in the plane orthogonal to the injection direction and the tunneling current is constant. Therefore, STT calculations based on the Slonczewski term are not physically realistic.

In this paper, the magnetization reversal process in CoFeB/MgO/CoFeB MTJs is theoretically investigated via an atomistic spin model combined with a model of spin accumulation, whose details can be found in the “Methods” section. To study the influence of temperature and size of CoFeB nanodots on switching time and reversal characteristics of magnetization, the atomistic model is used to construct the MTJ structure including the effect of interfacial magnetic properties^3,13,15^. For more realistic simulations of STT, the modified Simmons equation is used to determine the tunneling current flowing through the MTJ structure as a function of barrier thickness and relative angle of magnetization^21–24^. Interestingly, we find that the Slonczewski-based STT description in the often-used micromagnetic model tends to overestimate the torque and it causes non-coherent dynamics in smaller diameters than the spin accumulation based approach.

## Results

**Figure 1:**
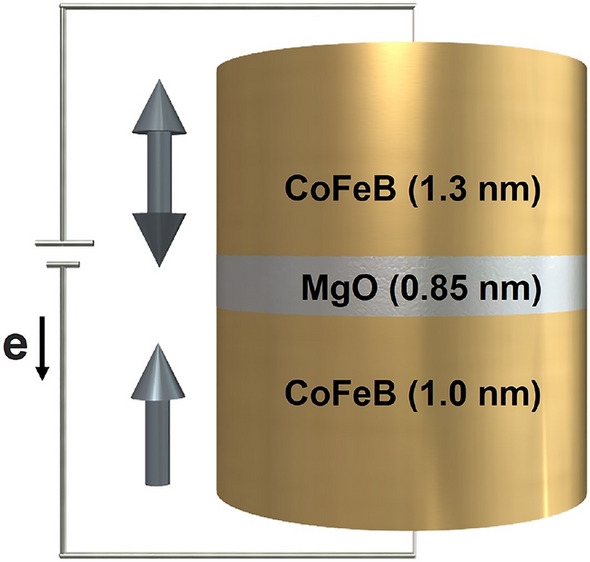
Schematic of the MTJ nanopillar of CoFeB (1.0 nm)/MgO (0.85 nm)/CoFeB (1.3 nm) with perpendicular magnetic anisotropy.

In this work, we focus on the investigation of thermally assisted magnetization switching in CoFeB–MgO based MTJs with comparatively low write energy for application in high-speed STT-MRAM. The MTJ nanopillar of CoFeB (1.0 nm)/MgO (0.85 nm)/CoFeB (1.3 nm) as illustrated in Fig. 1 is constructed at the atomistic level by using the vampire software package^25,26^. The model takes bulk and interfacial magnetic properties into account by considering a high anisotropy and high Gilbert damping constant at the interface of CoFeB/MgO. The magnetic properties and transport parameters of materials used in this paper are taken from the direct comparison with experiments in Refs.^11,13^. These parameters are shown in Table 1 and are defined in the section “Methods”.

**Table 1:** Magnetic parameters and transport properties of the CoFeB/MgO/CoFeB system.

Parameters	CoFeB (interface)	CoFeB (bulk)	MgO	Parameter name
$\alpha$	0.11	0.003	–	Gilbert damping
$J_{ij}$ (J/link)	$1.547\times 10^{-20}$	$7.735\times 10^{-21}$	–	Nearest-neighbours exchange energy
$k_u$ (J/atom)	$1.35\times 10^{-22}$	0	–	Uniaxial anisotropy energy
$\mu _s$ ($\mu _B$)	1.6	1.6	–	Atomic spin moment
$\beta$	0.56	0.56	0.11	Conductivity spin polarization
$\beta '$	0.72	0.72	0.14	Diffusion spin polarization
$\lambda _{\text {sdl}}$ (nm)	12	12	100	Spin diffusion length
${\mathrm {J_{sd}}}$ (eV)	0.1	0.1	0.01	*s*–*d* exchange energy
$\text {m}_\infty$ (MC/${\textrm{m}}^{3}$)	261.50	261.50	0.0	Equilibrium spin accumulation

**Figure 2:**
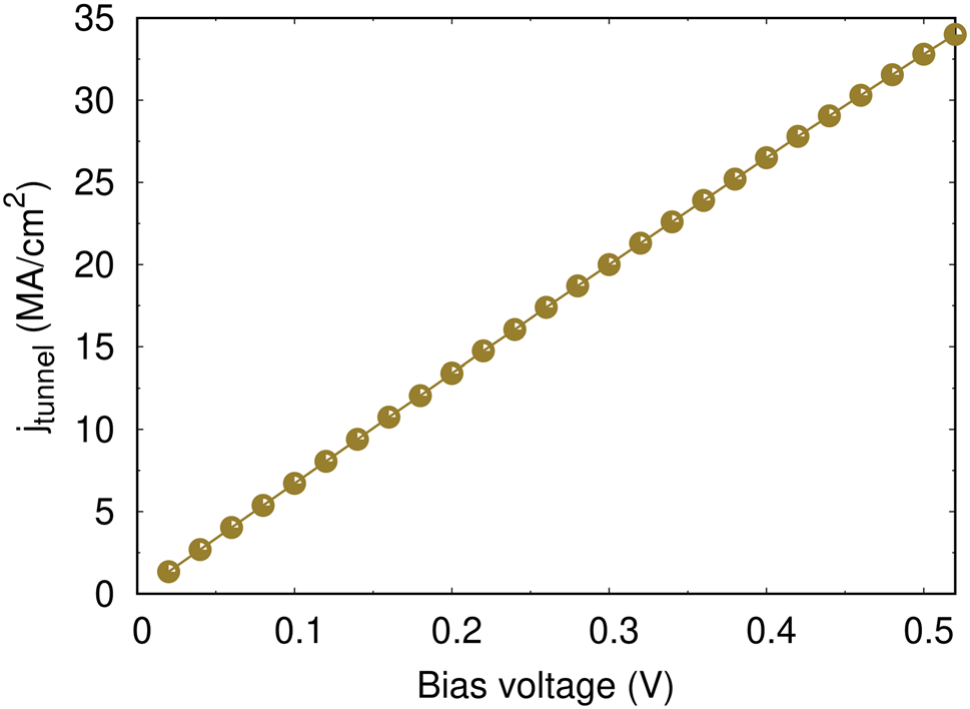
The average tunneling current density flowing through the MgO barrier of 0.85 nm thickness as a function of bias voltage calculated from the modified Simmons equation: The line of best fit is $j_{{\textrm{tunnel}}}=66.247$ V MA/cm$^2$ .

To study the reversal mechanism with the inclusion of thermal effects, the tunneling current flowing through the structure of CoFeB/MgO/CoFeB is first calculated using the modified Simmons equation given in Eq. (10) where the values of the parameters are taken from Ref.^24^: the Fermi energy relative to the conduction-band minimum in the contacts $E_F=2.2$ eV, the effective electron masses in the contact $m_{int}=0.3m_e$ and in the barrier $m_b =0.18m_e$ in units of the electron mass $m_e$, the majority–minority electron spin band splitting in the contacts $\delta =1.98$ eV and the potential barrier offset between the contact and the insulator $\varphi =1 \ eV$. The tunneling current density of parallel (P) and anti-parallel (AP) states flowing into the MTJ with barrier thickness of 0.85 nm are investigated by varying the bias voltage up to 0.5 V. Then the average tunneling current as a function of bias voltage, $j_{{\textrm{avg}}}=(j_{{\textrm{P}}}+ j_{{\textrm{AP}}})/2$, is calculated as shown in Fig. 2. This shows that tunneling current density has a direct relationship with the bias voltage. Furthermore, it was found that different configurations of magnetization result in varying tunneling current density values. The parallel state allows electron spins to relatively easily tunnel through the thin MgO barrier.

### Size dependence of magnetization reversal process

**Figure 3:**
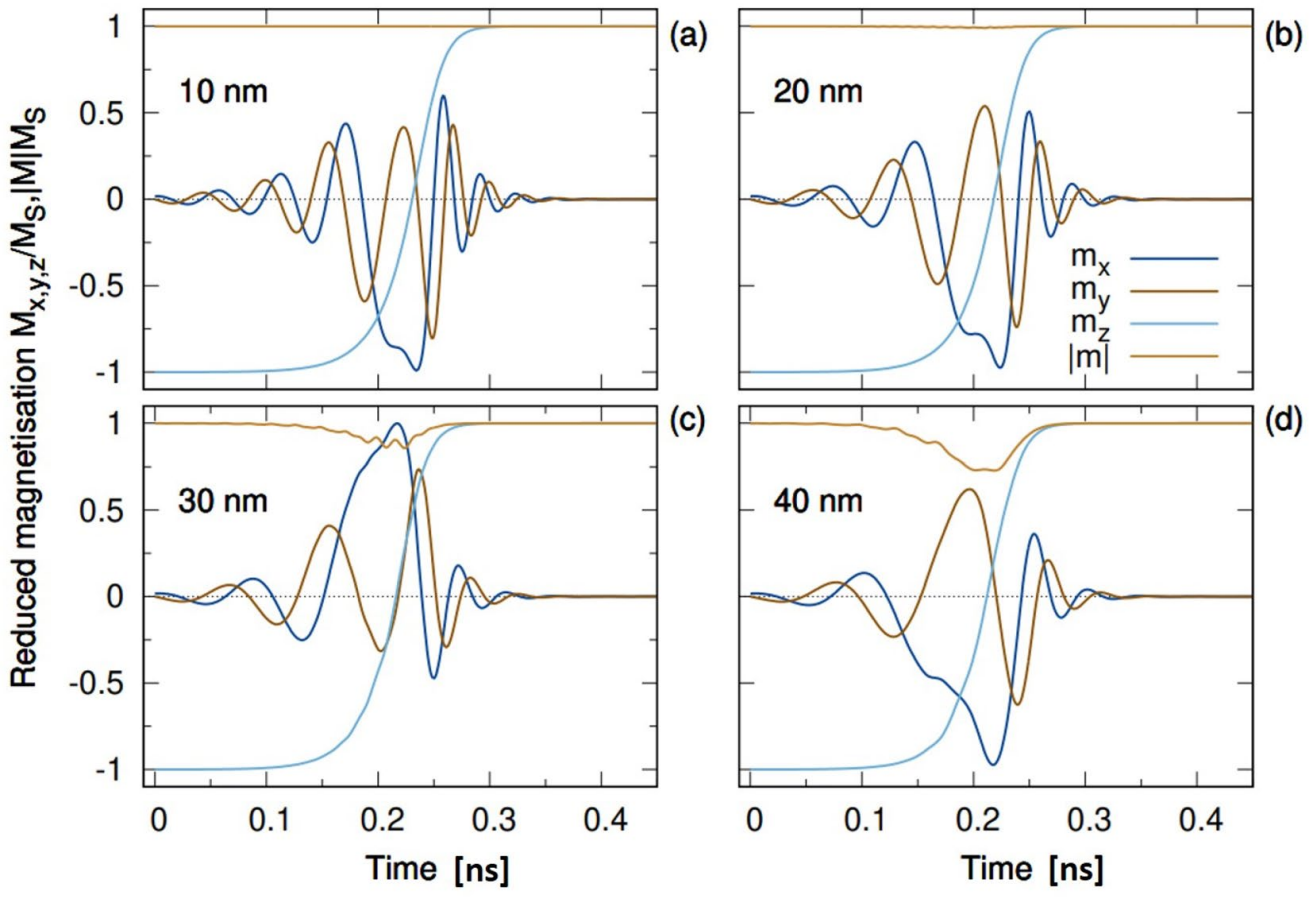
Dynamics of the magnetization in the free layer of the MTJ with different diameters, showing the evolution of the reversal mechanism for different MTJ diameters. For larger sizes the reversal becomes incoherent due to the partial formation of a domain during switching.

In this section, the effect of the diameter of the CoFeB-based MTJ on the magnetization dynamics is initially investigated at $T = 0$ K. We perform atomistic simulations of MTJs with different diameters of 10, 20, 30 and 40 nm by applying the bias voltage of 150 mV, resulting in a tunneling current density of 10 MA/cm$^2$ . This allows us to make a direct comparison with the results obtained in the previous work discussed in Ref.^14^, which is based on an atomistic Slonczewski approach with layer-resolved STT coefficients and where the same MTJ system parameters as here are used. The magnetization reversal process can be characterized by the time dependence of the perpendicular (*z*) component of magnetization, which can be divided into 2 time periods: the transient time and the reversal time. The transient time is the time taken to reverse the magnetization direction from its initial position until the *z* component of the magnetization is reduced by 10$\%$. The reversal time is defined as the duration from the transient time to the time where the z-component of the switched magnetization reaches − 90$\%$ of saturation. The sum of the transient and reversal times yields the overall switching time.

As shown in Fig. 3, the magnetization reversal can be observed to occur in the sub-nanosecond regime. The magnetization reversal behavior significantly depends on the size of the nanodot. Non-uniform magnetization, characterized by a reduction in the magnetization modulus during reversal is slightly noticeable in MTJ nanodots with diameter as small as 20 nm and is clearly present for larger diameters of 30 and 40 nm. Interestingly, our results show shorter transient and reversal times compared with those of the results in Ref.^14^, where, at the current density of 10 MA/cm$^2$ it takes the magnetization nearly 2 ns to fully switch and the reversal is coherent. On the other hand, for larger current densities the Slonczewski approach yields similar dynamical behavior to those obtained here with the spin accumulation model. Despite the similarities, for the diameter of 20 nm, close to the single domain size, the Slonczewski-based STT yields a non-coherent dynamics differently from the results presented here. This can be explained by considering that the tunneling current of the Slonczewski technique with layer-resolved STT coefficients does not take into account the influence of the barrier thickness, and the spin current is assumed to be fully polarized by the PL. There the field-like and damping-like torque coefficients of the Slonczewski model are parameterized and to do so assumptions were taken, as described in Ref.^14^. The comparison suggests that the Slonczewski model overestimates the effective field acting on the FL for a similar torque magnitude, thus allowing for non-coherent dynamics at smaller diameters. In fact the torque was applied uniformly to the whole in-plane surface of the FL, in contrast to the approach employed here using a spin transport model based on the spin accumulation. The comparison also highlights that larger current densities are required with the Slonczewski-based approach to induce a similar torque magnitude.

**Figure 4:**
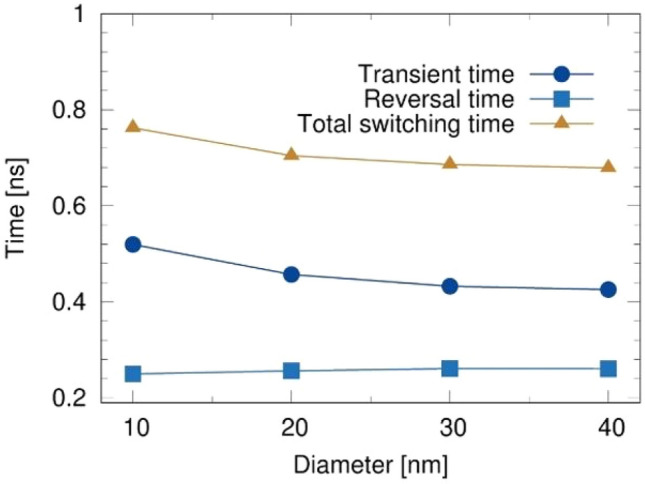
Magnetization reversal time in free layer of MTJ with different diameters injected by charge current density of 10 MA/cm$^2$ at $T=0$ K. The reversal time is dominated by the transient time which relates to the lower demagnetizing field for the small diameter MTJs, while the reversal time is not strongly dependent on the diameter.

As the diameter increases, the effective anisotropy of the CoFeB-perpendicular MTJ decreases due to the increasing demagnetization field contribution and incoherent reversal is more likely to occur. Thus the magnetization is more easily reversible as evident from the total switching time in Fig. 4. This is consistent with previous studies which reported the decrease of effective anisotropy field ($H^k_{{\textrm{eff}}}$)^27,28^ and energy barrier^29^ due to an increase of the in-plane contribution of the demagnetization energy with increasing disk diameter. This also influences the frequency of precessional motion of magnetization. For the large current density of 10 MA/cm$^2$ , the total switching time dependence on the in-plane dimensions of structure is dominated by the transient time: the reversal time is unaffected by size of MTJ-nanodot.

**Figure 5:**
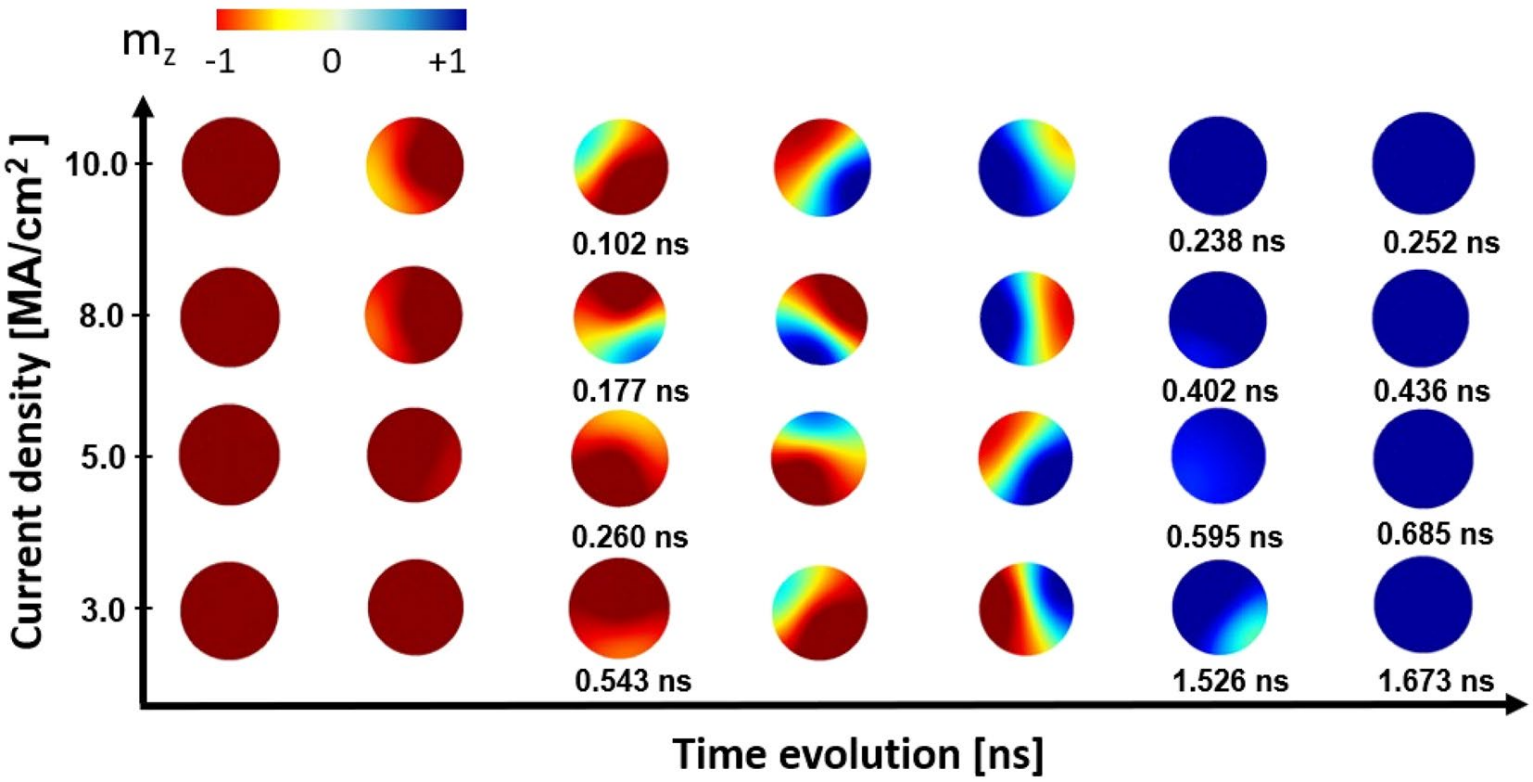
Visualization of the magnetization reversal of the FL magnetization of CoFeB/MgO/CoFeB MTJs with diameter of 30 nm for different injected current densities at 0 K. The color palette describes the z-component of the magnetization (red $=-z$, blue $=+z$, green = in-plane).

**Figure 6:**
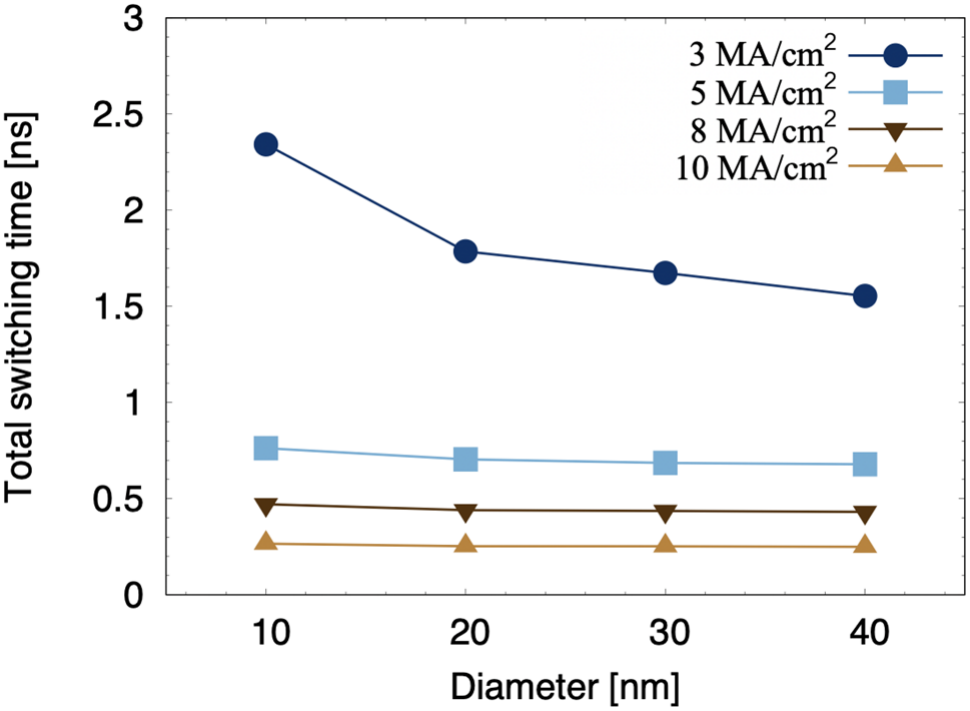
Total switching time in CoFeB/MgO/CoFeB MTJs as a function of the diameter for different current densities at 0 K.

The reversal of magnetization inside the MTJ structure of the STT-MRAM is controlled by the density of the current injected into the structure. Thus, in the following we focus on the dependence of the magnetization dynamics on the strength of the applied current. The results for the MTJ structure of diameter 30 nm is presented for applied voltages between 45 and 150 mV, where the application of these voltages results in an injected current density varying between 3 MA/cm$^2$ and 10 MA/cm$^2$ . As shown in Figs. 5 and 6, the reversal becomes faster as the injected current density increases. Increasing current densities results in increasing STT acting on the magnetization in the free layer. Fast magnetization reversal in just 0.685 ns is made possible by current densities exceeding 5 MA/cm$^2$ , which also significantly increase the precessional frequency of the magnetization originating from STT. The switching dynamics is incoherent with the magnetization reversal that evolves with nucleation of a reversed region at the disk edge which propagates through the disk, as shown in Fig. 5. The snapshots reveal also that the wall is asymmetric, with one edge broader than the other. This is due to the combination of the rotational dynamics of the in-plane magnetization and the fact that the torque acts in opposite directions at the two sides of the nucleated region. Since the nucleation is driven by the edge, we observe a non-symmetric curvature of the wall.

**Figure 7:**
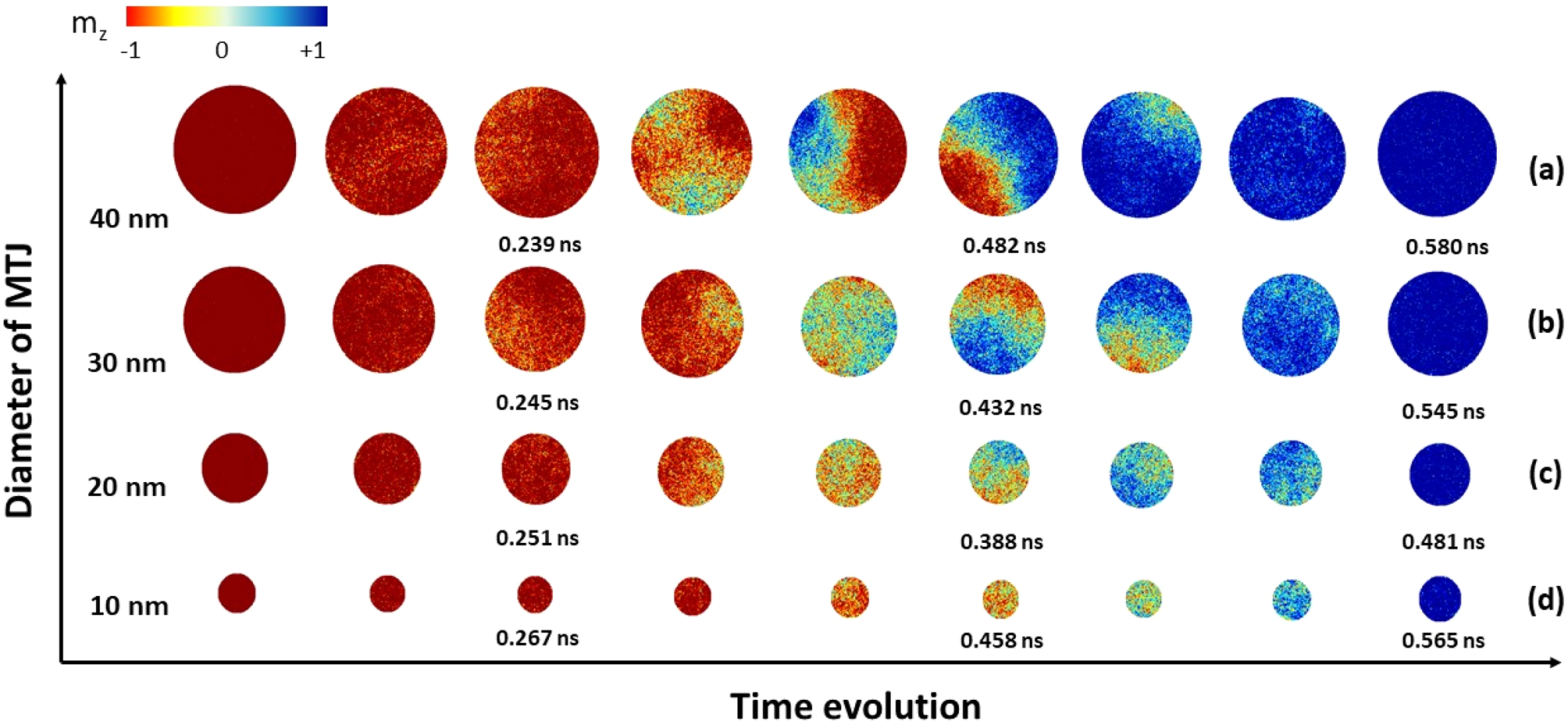
The time evolution of the out-of-plane (*z*-) component of the FL magnetization in MTJs with different diameters at 300 K for a injected current density of 5 MA/cm$^2$ .

Interestingly, the total switching time of the FL magnetization becomes size-independent for high current density as shown in Fig. 6. The reversal time is a characteristic of the system and depends mainly on the injected current density and temperature. The transient time instead has a dependence on the system size, in analogy with the coercive field. At high current densities the transient time is minimized with the reversal taking off within picoseconds from the initial excitation. Then the total switching time is determined by the reversal time. As a result, it is observed that the size of the MTJ and the density of the injected current have a significant impact on the magnetization reversal process, especially at low current density. Thus, to design CoFeB-based MTJ for the application in MRAMs with high operating speed and scalability, the density of injected current should be higher than 3 MA/cm$^2$ based on the results at zero temperature. Typically, the devices are operated at ambient temperature where the magnetization reversal behavior may differ from the athermal scenario and thermal fluctuations might relax some of the requirements. Our next interest therefore is the study of the impact of temperature.

### Influence of temperature on magnetization reversal

Here, the magnetization reversal mechanism in the MTJ structure with different diameters is examined at various temperatures up to 300 K. The effect of temperature, represented as a stochastic field, is included in the effective field in the LLG equation as described in “Methods”. Due to the finite size effect and the influence of temperature, we perform 20 independent relaxation simulations for each studied case to calculate the mean and variance of the switching time. The stochastic thermal field in each simulation is generated by a different set of pseudorandom numbers to ensure each simulation is statistically independent. In order to better understand the impact of finite size on magnetization switching in non-zero temperature scenarios, CoFeB-nanodots of various diameters are studied at 300 K. Following the results in Fig. 6, a charge current with a density of 5 MA/cm$^2$ is injected into the system, since this current density results in fast magnetization reversal with a switching time of less than 1 ns at zero temperature. Snapshots of the reversal at different times are shown in Fig. 7.

In comparison to the athermal scenario, where the incoherent reversal occurs in systems with diameters bigger than 20 nm, the magnetization reversal process at non-zero temperature is different. Thermal fluctuations are such that even at low current density the reversal is not fully coherent. The appearance of incoherent reversal can be seen starting from 20 nm and it becomes clearly observed in the CoFeB-nanodot system with large diameter. An important consequence is that while at 0 K, or low temperature, disks with a smaller diameter are fully coherent and characterized by a transient time that increases when reducing the diameter, at finite temperature it becomes easier to initiate the reversal. In analogy with what occurs in field driven reversal^13^, we can understand this by considering that on the one side thermal fluctuations make nucleation sites at the edge of the disk available, where spins are more loosely coupled. On the other, temperature is responsible for a reduction in the effective anisotropy and at very small diameters the disk approaches the superparamagnetic limit and leads to thermally instability. Thus thermal fluctuations make it possible to excite spins at the edge of the disk even at diameters of 20 nm, with the consequence that reversal becomes incoherent. It follows that with increasing temperature the transient time, which we can consider as the coercive field in field driven dynamics, is reduced resulting in a faster switching.

Figure 7 shows snapshots of the magnetization of the FL when a current density of 5 MA/cm$^2$ is injected into the system at 300 K for different diameters. The incoherent behavior can be clearly observed starting from a diameter of 20 nm as well as the rotational behavior of the in-plane components of the magnetization. An expected effect of temperature is an increase in the domain wall size, as clearly visible when comparing the snapshots for the 30 nm nanodot with those in Fig. 5. In a smaller diameter system, where the reversal remains mainly coherent, thermal fluctuations are manifest as a blurring of the rotating magnetization. Analogously to our findings for the case at 0K, a larger nominal injected current is required when employing the Slonczewski description of the STT to achieve similar dynamics. However, the results obtained with the spin accumulation model never reveal cases where the FL reverses via demagnetization associated with a suppression of coherent rotational mode, a behavior accompanied by the formation of a metastable vortex and antivortex spin structure. Rather, we always observe the nucleation of a reversed magnetized region that propagates through the disk. Such a difference with the results obtained with the Slonczewski-based approach at finite temperature suggests that indeed modeling STT via effective fields may overestimate the torque acting on the system and excite non-linear magnetization dynamics that would not be excited otherwise.

**Figure 8:**
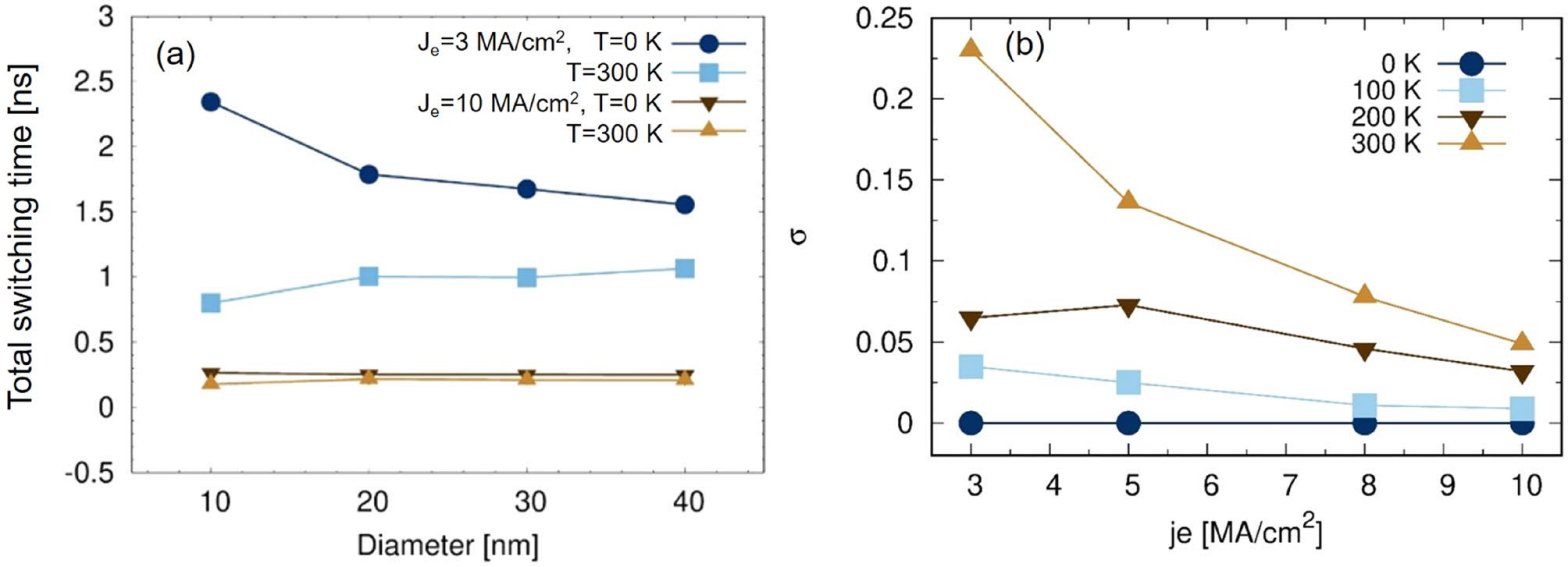
(**a**) Effect of finite size and temperature on the total switching time of magnetization in the free layer of CoFeB–MgO–CoFeB system by injecting the charge current density of 5 MA/cm$^2$ (**b**) the standard deviation of the calculated switching time as a function of current density for different finite temperatures for a MTJ diameter of 30 nm.

We finally investigate the effect of finite temperature on the switching time. In Fig. 8a we plot the total switching time as function of nanodot diameter at 0 K and at room temperature for low and high current densities, 3 and 10 MA/cm$^2$ respectively. At 0 K, as discussed above, the total switching time increases for smaller diameter. At 300 K we observe the opposite trend: a decrease for smaller diameters. At finite temperature small systems have a reduced thermal stability due to the reduced volume and finite size effects which reduce the value of anisotropy. The size dependence is more marked for low current densities as one would expect. Nevertheless, it is interesting to observe that the difference in the switching time between 0 K and 300 K decreases with the current density. As the current density increases the transient time decreases until the reversal starts as soon as the torque is applied. The dynamical behavior is then dominated by the reversal time, which is a characteristic of the system and depends on the current density and only weakly on the temperature. Instead, lower current densities require longer times to induce the reversal, hence the difference.

The standard deviation $\sigma$ of the total switching time as a function current density magnitude and temperature is presented in Fig. 8b for a diameter of 30 nm. In the absence of structural defects and pinning sites the only possible origin of a distribution is the thermal field. Hence, $\sigma$ provides a measure of thermal effects on the magnetization dynamics. It follows that if there is no temperature there is no distribution. As the temperature increases so does $\sigma$, approaching 0.25 ns for the lowest current density at 300 K. $\sigma$ tends rapidly towards values below 0.05 ns as the current density increases and exhibits a weak size dependence (not shown here) for nanodot diameters larger than 10 nm. At elevated temperatures the smallest diameters tend towards thermal instability; as a consequence the distribution of transient time is larger affecting the total switching time $\sigma$. This indicates that the device investigated in the present work is not suitable for applications below the 20 nm node, and improvements to either the structure or the material parameters would be necessary. This weak size dependence is indication of the intrinsic nature of the thermal fluctuations and marks the stochastic character of the STT reversal. $\sigma$ is an irreducible contribution to the dispersion in switching times and must be considered in device design. Moreover, the fact that the switching time tends towards a constant value for diameters larger than 20 nm is indicative of a domain wall mediated reversal mechanism, as confirmed by inspecting the snapshots of the magnetization in Fig. 7.

The switching time calculations show very good agreement with the results presented in Ref.^14^. We observe some numerical differences which tend to be more marked at weaker current densities. As noted already both at 0 K and at finite temperature, the Slonczewski-based STT description tends to overestimate the torque. This may be hidden when the reversal takes a few hundreds of picoseconds, while it emerges for slower processes with lower current densities. It might appear surprising on the other hand that the agreement for the switching time distribution is also quantitative. However, despite the different approach to the STT, thermal fluctuations are treated in the same way. Since both systems are ideal, thermal effects will be similar.

From our investigations it emerges that the magnetization reversal process in CoFeB-based MTJ strongly depends on the injected current density, size of the nanodot and the temperature. For real application of STT-MRAMs, MTJs with lateral size smaller than 20 nm, fast switching in the sub-nanosecond regime and low energy consumption are required. Our results show that more sophisticated and complex systems need to be considered to achieve such a goal. Nonetheless our study and approach can be used as guidance to develop high-performance MTJs for desired applications.

## Discussion and conclusions

We have made a theoretical study of the magnetization switching of CoFeB-based MTJ driven by the STT effect. An atomistic model coupled with a spin transport model is used to investigate the magnetization reversal behavior in MTJs with different diameters at 0 and 300 K. The non-uniform tunneling current expressed in terms of the barrier thickness and the bias voltage is considered by employing the modified Simmons equation. For the athermal case, the reversal is coherent below 20 nm, whereas a domain wall nucleation and propagation is involved in the reversal process for larger MTJ sizes. In addition, the switching time is weakly size-dependent for the highest current densities. At temperatures relevant for applications the magnetization reversal is thermally driven and the incoherent mode can be excited in smaller diameters. Thermal fluctuations are responsible for the stochastic character of STT-switching dynamics visible in the onset of the reversal and measurable as the distribution of the switching time. The switching time significantly decreases with increasing temperature and is characterized by a smaller distribution as the current density increases. This study provides improved understanding of magnetization switching via the influence of STT and points out factors important for the design of MTJs for various applications. We also compare our results based on a spin transport model with the case where the STT is described via external fields in a Slonczewski-like approach. The results tend to agree qualitatively, however it emerges that the latter tends to overestimate the torque acting on the magnetization a factor which is accentuated at finite temperature.

## Methods

### Atomistic model

The dynamical behavior of the spin system including the effect of STT is obtained by integrating the stochastic Landau–Lifshitz–Gilbert equation of motion (LLG)^26,30^ given by:

1 $$\begin{aligned} \frac{\partial {{\textbf{S}}}}{\partial t}= -\frac{\gamma }{(1+\alpha ^2)} \left( {{\textbf{S}}} \times {{\textbf{B}}}_{{\textrm{eff}}}\right) -\frac{\gamma \alpha }{(1+\alpha ^2)} [{{\textbf{S}}}\times ({{\textbf{S}}} \times {{\textbf{B}}}_{{\textrm{eff}}})] \end{aligned}$$

where ${{\textbf{S}}}$ is the normalized atomic spin vector, $\gamma$ is the absolute value of gyromagnetic ratio, $\alpha$ is the intrinsic damping constant, representing coupling to the heat bath at the atomic level and ${{\textbf{B}}}_{{\textrm{eff}}}$ denotes the effective field acting on each atomic spin. ${{\textbf{B}}}_{{\textrm{eff}}}$ is determined from the energy contributions of the magnetic system described by the following extended Heisenberg spin Hamiltonian $\mathcal {H}$^26^:

2 $$\begin{aligned} \mathcal {H}=- \sum \limits _{i < j}{{J_{ij}}{{\mathbf{{S}}}_i}\cdot {{\mathbf{{S}}}_j}}-{k_{\textrm{u}}} \sum \limits _{i}({{\mathbf{{S}}_i}}\cdot {\mathbf{{e}}})^2- \sum \limits _{i}{\mu _{\textrm{s}}^i\textbf{S}}_i \cdot {\mathbf{{B}}_{\textrm{app}}}-J_{\textrm{sd}} \textbf{m} \cdot {{\textbf{S}}}_i. \end{aligned}$$

$J_{ij}$ is the nearest neighbor exchange integral between spin sites *i* and *j*, ${{\textbf{S}}}_{i,j}$ is the normalized spin vector on site (*i*, *j*), $k_{\textrm{u}}$ is the uniaxial anisotropy constant, ${{\textbf{e}}}$ is the easy axis unit vector and $\mu _{\textrm{s}}^i$ is the magnitude of the spin moment on site *i*. The first term of the spin Hamiltonian represents the exchange energy. The second and third terms are the anisotropy energy and the Zeeman energy associated with an external field, respectively. The final term describes STT arising from the $s-d$ exchange interaction between the spin accumulation ($\textbf{m}$) and the local spin moment, where *s* electrons represent the former and *d* electrons the latter contribution.

In addition to the contributions given in the the spin Hamiltonian in Eq. (2), the demagnetizing field and thermal fluctuations are taken into account in the model. The demagnetizing field is added directly to ${{\textbf{B}}}_{{\textrm{eff}}}$ using a modified macro-cell approach^31^. In this method the system is discretized into cubic macro-cells, treated as regions with uniform magnetization, and the dipolar interaction between these is calculated. The dipolar field for a macro-cell *k* is given by:

3 $$\begin{aligned} {\textbf{B}_{\textrm{dip,}k}} = \frac{{{\mu _0}}}{{4\pi }}\sum \limits _{l \ne k} {\left[ {\frac{{3({{{\varvec{\mu }}}_l} \cdot \hat{{{\textbf{r}}}}_{kl})\hat{{{\textbf{r}}}}_{kl} - {{{\varvec{\mu }}}_l}}}{{|r_{kl}{|^3}}}} \right] }, \end{aligned}$$

where $\varvec{\mu }_l$ is the vector describing the magnetic moment of the macro-cell *l* containing $n_{{\textrm{atom}}}$ spins:

4 $$\begin{aligned} {{\varvec{\mu }}_l} = \sum \limits _{i =1}^{{n_{\textrm{atom}}}} \mu _{\textrm{s}}^i{{\textbf{S}}}_i. \end{aligned}$$

$\mu _0$ is the permeability of free space, $r_{kl}$ is the distance between macro-cell *k* and *l*, $\hat{{{\textbf{r}}}}_{kl}$ is the corresponding unit vector. This approach, based on the work of Bowden^32^, accounts for the contribution within each cell by explicitly computing the interaction tensor utilising atomistic coordinates. The tensor, given by the summation in Eq. (3), comprises of two contributions: (a) the *inter macro-cell* interaction of the atomic moments within a macro-cell with the atomic moments in another cell, and (b) the *intra macro-cell* interaction between spins belonging to the same macro-cell. This approach works independently of the shape of the macro-cell and thanks in particular to the intra-macro-cell term it achieves an accurate description of the dipolar contribution for surfaces and irregular shaped regions. We remark that in such a macro-cell approach all the spins within a macro-cell experience the same dipole field, that is all spins *i* within macro-cell *l* will be subjected to the same $\textbf{B}_{\textrm{dip,}l}$. Provided the magnetization is uniform within the cell ($V = 1$ nm$^3$) this is a good approximation.

Finite temperature effects are described via the inclusion of a stochastic field ${\mathbf{{B}}^i_{\textrm{th}}}$ in the effective field, taking into account dissipation effects and exchange of energy with the heat bath. Following Brown’s approach^33^, referred to as Langevin dynamics, the thermal field at the nanosecond time scale can be described as a white noise term^34^. In this limit ${\mathbf{{B}}^i_{\textrm{th}}}$ is described by a Gaussian distribution in 3 dimensions, $\varvec{\Gamma }(t)$, and the first two statistical moments of the distribution are obtained from the fluctuation-theorem and Fokker-Planck equation as follows:

5 $$\begin{aligned} \left\langle {\mathbf{{B}}^i_{\textrm{th}}(t)} \right\rangle&= 0 \nonumber \\ \left\langle {\mathbf{{B}}^{i,b}_{\textrm{th}}(t) \mathbf{{B}}^{j,b}_{\textrm{th}}(t')} \right\rangle&= \frac{2\alpha {k_B}T}{{\mu _s}|\gamma |} {\delta _{ij}}{\delta _{ab}}\delta (t - t') \,. \end{aligned}$$

*i*, *j* label the atomic spins on the respective sites, *a*, *b* are the Cartesian components of $\textbf{B}_{\textrm{th}}$, $t,t'$ are the time at which the fluctuations are evaluated and T is the system temperature, $k_B$ is the Boltzmann constant and the Gilbert damping $\alpha$ serves to couple the spin system with the heath bath. ${\delta _{ij}},{\delta _{ab}}$ are Kronecker delta, and $\delta (t - t')$ is the Dirac delta function. The resulting thermal field $\mathbf{{B}}_{\textrm{th,i}}$ acting on site is

6 $$\begin{aligned} \mathbf{{B}}_{\textrm{th,i}} = \Gamma (t)\sqrt{\frac{2\alpha k_BT}{\gamma \mu _s\Delta t}}, \end{aligned}$$

where $\Gamma (t)$ is extracted from a normal distribution.

The net local field $\textbf{B}_{\textrm{eff,}i}$ acting on each atomic spin *i* that accounts for interactions within the system and the effect of temperature is given by:

7 $$\ {{\textbf{B}}_{\textrm{eff},i}}= - \frac{1}{{\mu _s}^{i}}\frac{{\partial {\mathcal {H}}}}{{\partial {{{\textbf{S}}}_{i}}}} + {{\textbf{B}}_{\textrm{dip},i}}+ {\mathbf{{B}}_{{\textrm{th,i}}}}.$$

where *i* in ${{\textbf{B}}_{\textrm{dip},i}}$ refers to the dipolar field acting on the atomic spin *i*. The integration of the LLG Eq. (1) is performed numerically using a Heun scheme^26^.

### Spin-transfer torque

The contribution of STT originating from the spin accumulation can be calculated in a basis coordinate system where the unit vector $\hat{\textbf{b}}_1$ and $\hat{\textbf{b}}_{2,3}$ are parallel and perpendicular to the direction of local spin moment^35^. Following Zhang et. al.^36^ we separate the spin accumulation solution into longitudinal (${{\varvec{m}}_{\parallel }}$) and transverse (${{\varvec{m}}_{\bot }}$) contributions written as follows^35,37^:

8 $$\begin{aligned} {\textbf{m}_{\parallel }}(x)&= \left[ {{m}_{\parallel }}(\infty ) + \left[ {{m}_{\parallel }}(0)-{{m}_{\parallel }}(\infty ) \right] e^{- x/{\lambda _{\textrm{sdl}}}} \right] \hat{{\textbf{b}_1}} \nonumber \\ {\textbf{m}_{\bot , 2}}(x)&= 2e^{-k_1x}\left[ u \cos (k_2x)-v \sin (k_2x) \right] \hat{{\textbf{b}_2}} \nonumber \\ {\textbf{m}_{\bot ,3}}(x)&= 2e^{-k_1x} \left[ u \sin (k_2x)+v \cos (k_2x) \right] \hat{{\textbf{b}_3}}, \end{aligned}$$

where $(k_1 \pm ik_2) = \sqrt{\lambda _{\textrm{sf}}^{-2} \pm i \lambda _{J}^{-2}}$, ${\lambda _{\textrm{sdl}}}$ is the spin diffusion length, ${m}_{\parallel }(\infty )$ is the spin accumulation at equilibrium, the spin-flip length is defined as ${\lambda _{\textrm{sf}}} = \sqrt{2{D_0}{\tau _{sf}}}$ and ${\lambda _{\textrm{J}}}$ is the spin-precession length. the unknown variables *u*, *v* and $m_{\Arrowvert }(0)$ can be determined by imposing continuity of the spin current ($j_m$) across interfaces, with $j_m$ given by:

9 $$\begin{aligned} {\textbf{j}_m}(x) = \beta {j_{\textrm{tun}}}{\textbf{M}} - 2{D_0}\left[ {\frac{{\partial {{\textbf{m}}}}}{{\partial x}} - \beta \beta '{{\textbf{M}}}\left( {{{\textbf{M}}} \cdot \frac{{\partial {{\textbf{m}}}}}{{\partial x}}} \right) } \right] \end{aligned}$$

where $\beta$ and $\beta '$ are the spin polarization parameters of the material, $D_0$ is the diffusion constant and ${j_{\textrm{tun}}}$ is the density of tunneling current flowing through the MTJ structure. The tunneling current can be solved by applying the modified Simmons equation^21–24,38^ given by:

10 $$\begin{aligned} j_{\textrm{tun}} = J_0 [J^\uparrow +J^\downarrow ], \end{aligned}$$

where $J_0$ is the tunneling current density calculated from the original Simmons equation expressed in terms of the barrier thickness and the bias voltage and $J^{\uparrow (\downarrow )}$ is the tunnelling current density of up (down) spin as follows:

$$\begin{aligned} J_0=\frac{e}{4\pi ^2\hbar (S\zeta )^2} \left[ \left( \phi -\frac{eV}{2} \right) e^{-A \sqrt{\phi -\frac{eV}{2}}} -\left( \phi +\frac{eV}{2}\right) e^{-A \sqrt{\phi +\frac{eV}{2}}} \right] \end{aligned}$$

with

$$\begin{aligned} J^{\uparrow (\downarrow )}= \frac{16{k}^{\uparrow (\downarrow )}_{\textrm{FM1}}\xi ^2}{\xi ^2+({k}^{\uparrow (\downarrow )}_{\textrm{FM1}})^2} \left[ \frac{{k}^{\uparrow (\downarrow )}_{\textrm{FM2}}\cos ^2(\theta /2)}{\xi ^2+ ({k}^{\uparrow (\downarrow )}_{\textrm{FM2}})^2} +\frac{{k}^{\uparrow (\downarrow )}_{\textrm{FM2}}\sin ^2(\theta /2)}{\xi ^2+ ({k}^{\uparrow (\downarrow )}_{\textrm{FM2}})^2} \right] . \end{aligned}$$

The spin-polarized electron momentum of ferromagnetic layers are given by:

$$\begin{aligned} {k}^{\uparrow (\downarrow )}_{\textrm{FM1}}&= \frac{ \sqrt{2m_{int}(E_F-(\delta \mp \delta -eV)/2)}}{\hbar } \\ {k}^{\uparrow (\downarrow )}_{\textrm{FM2}}&= \frac{\sqrt{2m_{int}(E_F-(\delta \mp \delta +eV)/2)}}{\hbar } \end{aligned}$$

and

$$\begin{aligned} A= \frac{2 \zeta S {\sqrt{2m_e}}}{ \hbar },\quad \xi = \frac{m_{int}}{m_b}\frac{\sqrt{2m_b\varphi }}{\hbar }, \end{aligned}$$

where *S* is the thickness of insulating film, $\varphi$ is the tunnel barrier height, *V* is the electric potential, $m_e, e$ are the electron mass and charge respectively, $\zeta$ is an empirical constant, $\theta$ is the relative angle between the magnetization in the PL and the FL, $E_F$ is the Fermi energy, $\delta$ is the band splitting between the majority and minority spins, and $m_{int}$ and $m_b$ are the effective electron masses in the interface and the barrier respectively.

## Data Availability

The datasets generated during and/or analysed during the current study are available from the corresponding author on reasonable request.
